# Immunoception: the insular cortex perspective

**DOI:** 10.1038/s41423-023-01051-8

**Published:** 2023-06-29

**Authors:** Asya Rolls

**Affiliations:** Department of Immunology, Department of Neuroscience, Technion, Israel Institute of Technology, Haifa, Israel

**Keywords:** Inflammation, brain, sympathetic, Neuroimmunology, Immunology

## Abstract

To define the systemic neuroimmune interactions in health and disease, we recently suggested immunoception as a term that refers to the existence of bidirectional functional loops between the brain and the immune system. This concept suggests that the brain constantly monitors changes in immune activity and, in turn, can regulate the immune system to generate a physiologically synchronized response. Therefore, the brain has to represent information regarding the state of the immune system, which can occure in multiple ways. One such representation is an immunengram, a trace that is partially stored by neurons and partially by the local tissue. This review will discuss our current understanding of immunoception and immunengrams, focusing on their manifestation in a specific brain region, the insular cortex (IC).

## Introduction

In recent years, there has been a growing body of evidence suggesting that the brain continuously monitors the state of the immune system and can modify immune responses based on this acquired information. This bidirectional communication, which we term immunoception [1], is based on the classical neuroscience concept of interoception [2, 3]. The insular cortex (IC), a key area for forming interoception [2], has been shown to store immune representations. Therefore, this review focuses on the involvement of the IC in immune representation and regulation. First, it will cover the evidence supporting the insular cortex’s involvement in immune representation and regulation and then it will discuss the potential communication pathways between the insular cortex and its peripheral immune counterparts. In addition, the concept of immunengram, which refers to the physical trace of immune-related activity, will be discussed, along with some open questions in the field.

## From interoception to immunoception

### Interoception

The term “interoceptive” was first introduced by Sherrington in 1906 to refer to senses that convey information regarding the internal state of the body, in contrast to exteroceptive sensing, which refers to sensitivity to stimuli outside the body [2].

Over time, the concept has evolved to encompass the process by which the nervous system receives, integrates, and interprets sensory information from within the organism. This includes physiological signals such as heart rate, breathing, hunger, and pain [3, 4], which are continuously monitored through the detection of pressure changes, temperature, contraction and stretching of the viscera, and levels of nutrients, gases, toxins, and chemicals within the body [4]. These stimuli are detected by chemoreceptors, osmoreceptors, glucoreceptors, mechanoreceptors, and humoral receptors [2].

Interoceptive information is not limited to physiological inputs and includes limbic and cognitive inputs. As a result, interoception is crucial to our ability to perceive and regulate our internal states and emotions and is involved in a wide range of physiological and psychological processes [5]. The brain integrates this stream of sensory signals at both conscious and unconscious levels and adjusts physiological processes to maintain allostasis, a form of dynamic homeostasis [6]. In this way, interoception also underlies the formation of urges, feelings, drives, adaptive responses, and cognitive and emotional experiences.

### Immunoception

The immune response is fundamental to maintaining the organism’s integrity and is activated in response to external challenges and internal deviations from homeostasis. The immune response was also shown to be involved in regulating metabolic processes [7], stress reactions [8], and even cognitive and emotional processes [9]. Hence, the activity of the immune system can be considered an indicator of the organism’s state, providing information essential for generating an interoceptive image of the body. This image is generated by the brain via a range of inputs acquired, at least in part, by the sensory nervous system.

Such neuroimmune interactions require a unique analysis framework that will allow capturing of the immune complexity by the nervous system. The immune response can manifest on different scales (systemic or local), involve different agents (diversity of cytokines and cellular responses), and impact various tissues and organs. Immune system activity can also affect other interoceptive functions, such as metabolism, temperature, and blood pressure. Hence, acquiring immune-related information requires the involvement of multiple components of the sensory system.

The complementary component of immunoception, is immune regulation by the nervous system, which also requires a unique set of modulatory agents that can be detected by immune cells directly or indirectly [10]. Examples include local neuronal secretion of neuropeptides, the receptors of which are expressed by immune cells [11, 12], modulation of blood vessel permeability [13] or mobilization of metabolic agents.

The definition of immunoception as the continuous bidirectional flow of information between the brain and the immune system entails forming a central representation of the organism’s immunological state by the brain. This central representation of the immune system is expected to be manifested across multiple brain modules that can encompass the complexity of the immune response. Indeed, it was shown that peripheral inflammation results in increased activity across the entire brain [14–16]. Here, we will focus on the IC, or insula, considered the “primary interoceptive cortex” [4] and highlight some of this region’s unique properties, positioning the IC as a key component in immunoception.

## The insular cortex

**Fig. 1:**
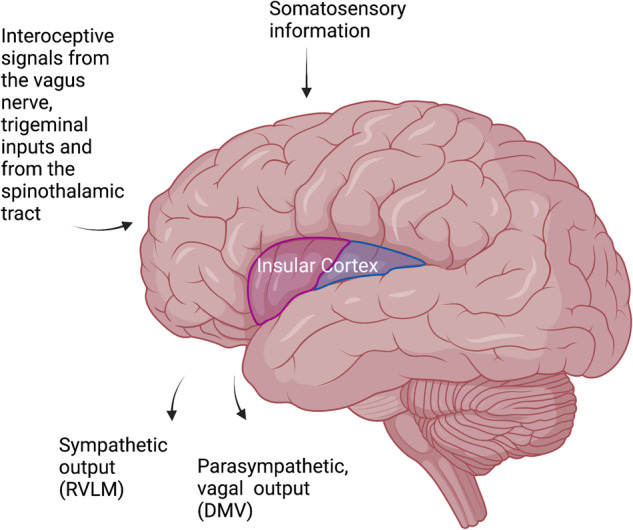
Insular cortex connections relevant to the immune response. The insular cortex integrates interoceptive and somatosensory inputs and can affect autonomic nervous system activity via the RVLM and DMV

The IC is known to play a key role in generating interoception [17], and its involvement in immune regulation has become increasingly apparent [18–22]. The IC is located deep within the lateral sulcus (or Sylvian fissure), which is a prominent groove on the lateral surface of the brain (Fig. 1). It is considered part of the cerebral cortex, the outer layer of the brain responsible for complex cognitive and sensory processing. The IC plays a vital role in a range of processes related to bodily and self-awareness [4], bodily sensations [23], emotions [24, 25], multisensory integration [26], and learning and memory [27]. It is involved in the integration and interpretation of interoceptive signals from the body, such as those related to hunger, thirst, and pain [17, 28, 29].

The IC is divided into three main regions: the anterior (aIC), mid (mIC), and posterior insula (pIC). In humans, the distinction between the anterior and posterior parts is determined by the central insular sulcus. Cytoarchitectural subregions in the IC are ordered from the dorsal to ventral cortex, known as the granular, dysgranular, and agranular. The granular insular cortex has a classical six-layered structure; in the dysgranular insula, layer 4 becomes thinner; and the agranular insula is tri-laminar, entirely lacking layer 4. The three subdivisions are strongly interconnected along the dorso-ventral and rostro-caudal axes [26].

In general, the anterior insula is involved in processing emotions, empathy, social cognition, and decision-making, while the mid and posterior insula is primarily involved in somatosensory processing or the processing of sensory information from the body [26], although both areas were shown to be involved in generating interoception [2, 3, 5, 30].

The IC is massively connected to the rest of the brain, and whole-brain inputs and outputs of the mouse IC were mapped using viral vectors in a very important study [31]. To characterize afferent connections onto IC neurons, the authors used cell-type specific monosynaptic rabies virus tracings, while adeno-associated viral (AAV) tracings were used to label efferent axons. It was shown that IC connectivity is characterized by strong projections to the striatum and reciprocal connections with diverse subregions of the amygdala and the thalamus. The IC subregions differ in their inputs and outputs: the pIC receives twice as many inputs from the sensory cortices compared to the other IC subdivisions, indicating that this area collects peripheral sensory and visceral inputs. The mIC and the aIC are strongly connected to the amygdala, which is not only involved in emotional regulation and memory but also has been shown to regulate immune activity [31]. Thus, functionally and anatomically, the IC serves as a hub that integrates bodily information with memory and emotional content to guide behavior and maintain homeostasis.

## Evidence for the involvement of the insular cortex in immune activity

It is now well established that the brain responds to changes in peripheral inflammation [14–16]. The most intuitive example is sickness behavior, in which physiological and behavioral processes, including sleep, appetite, mood, and cognition, are altered during inflammation or infection [32–36]. All of these functions are regulated by the brain, specifically by neuronal networks that were shown to be affected, at least in part, by cytokines [32–36]. The specific involvement of the IC in peripheral inflammation has been demonstrated through several lines of evidence: (1) imaging and activity mapping, (2) functional studies, and (3) anatomical evidence for connections between the IC and peripheral immune organs.

### Imaging and activity mapping

Imaging studies and activity mapping approaches, e.g., fMRI studies, revealed changes in IC activity during peripheral inflammation. Clearly, the IC is not the only brain area that responds, and a meta-analysis of 24 fMRI human studies found that the amygdala, hippocampus, hypothalamus, striatum, midbrain, brainstem, prefrontal and temporal cortices as well as the IC were all activated in response to peripheral inflammation [16]. Another imaging method is quantitative magnetization transfer (qMT) imaging, a magnetic resonance imaging technique that enables the quantification of changes in brain macromolecular density. Inflammation induces a rapid change in the brain microstructure within a discrete region of the IC, which is implicated in representing internal physiologic states, including inflammation [37]. The functional significance of this change in insular microstructure was demonstrated by correlation with inflammation-induced fatigue and fluorodeoxyglucose positron emission tomography imaging (FDG-PET), which revealed increased resting glucose metabolism within this region following systemic inflammatory challenge [37].

Disease states provide another line of supportive evidence, as patients with inflammatory bowel disease [38–42] or arthritis [43] were shown to have altered activity and connectivity profiles in the IC. Nevertheless, these studies are largely confounded by the fact that the IC is part of the pain matrix. Thus, the altered activity in this area may represent, as discussed below, the response to the pain, characteristic of these conditions.

Another approach that allows evaluating changes in brain activity during inflammatory reactions is c-Fos labeling. c-Fos is an immediate early gene expressed upon neuronal activation, and therefore, its expression is generally considered a marker for neuronal activity. Nevertheless, this approach can only be applied in animal studies, and not all active neurons manifest elevated c-Fos levels. Activation of c-Fos is often used as a proxy for identifying brain regions responding to a particular stimulus or condition, including immune challenges. Several studies have used c-Fos imaging techniques, such as immunohistochemistry or in situ hybridization, to investigate the brain’s response to immune challenges. For example, a study that examined the expression of c-Fos in response to lipopolysaccharide (LPS) found increased c-Fos expression in the hypothalamus, amygdala, and IC [21, 44, 45].

An extension of the c-Fos labeling approach is targeted recombination in active populations (TRAP) [46]. This approach is based on a recombination event between two transgenes, one expressing the tamoxifen-dependent recombinase CreERT2 from an activity-dependent IEG promoter, Fos, and the other expressing an effector gene, such as a fluorescent reporter, in a Cre-dependent manner. In the presence of tamoxifen, active cells expressing *cfos* are permanently tagged with a fluorescent protein. Thus, in TRAP mice, one can visualize the neurons that were active at a given time point, e.g., during peripheral inflammation. Using TRAP mice, we showed that during DSS-induced colitis [5], a model of peripheral colon inflammation, there is increased activity in the IC. This area was also active under another inflammatory model, zymosan-induced peritonitis. Interestingly, although in both these models we monitored the same brain region, the IC, the specific neurons activated in each inflammatory episode were different even when we tinduced he same type of inflammation (zymosan-induced peritonitis). Thus, collective evidence indicates elevated activity in the IC during inflammation, however, although these neurons are active, it does not prove that they have any functional relevance to the immune response.

### Functional evidence

Some of the most exciting evidence in support of a functional connection between the IC and immune activity comes from human studies [47]. Nevertheless, these are mainly correlative studies that are limited in their interpretation. For example, a study compared the frequency of hospital-acquired pneumonia, a major complication of stroke, in patients with left versus right hemispheric infarcts (controlled for age, gender, infarct size, dysphagia, and six other clinical variables) [48]. Hospital-acquired pneumonia was more frequent in patients with right hemispheric infarcts versus left hemispheric infarcts. This appears to be most relevant to the IC, as out of the 10 most infarcted regions, only the right insular cortex volume was different in patients with hospital-acquired pneumonia versus controls [48].

Even more striking are the effects on patients with stroke or other brain injury who also suffer from an autoimmune disease. For example, patients with arthritis who experienced a stroke were shown to have enhanced antigen-specific T-cell reactivity on the stroke-affected side of the body [49]. This effect was proposed to be mediated by changes in sympathetic activity [50]. Patients with minor stroke or poliomyelitis show weaker delayed-type hypersensitivity (DTH) responses on the paretic side [51, 52].

Although not specific to the IC, in patients with epilepsy, resections in the language-dominant hemisphere were correlated with reduced levels of lymphocytes, total T cells, and helper T cells. In contrast, resections in the language nondominant hemisphere correlated with increases in the same immune cells [53]. Accordingly, manipulating neuronal activity in either the left or the right hemisphere of the rodent brain was shown to result in opposing immunological reactions [46, 54–58], suggesting that the two hemispheres have distinct effects on the peripheral immune response.

Another line of evidence comes from psychiatric patients. Systemic inflammation and immune dysregulation have been considered risk factors in the pathophysiology of mood disorders, including bipolar disorder (BD). Conversely, neuroimaging studies have revealed that disrupted functional connectivity between the IC and sensorimotor areas is associated with elevated proinflammatory cytokine levels of IL-6 in BD [59].

fMRI studies in patients with rheumatoid arthritis treated with TNF-α-neutralizing therapies revealed changes in brain activity in multiple areas, including the IC, within 24 h after treatment. This effect was evident before any effect on the joint, measured by clinical and laboratory markers of inflammation, such as joint swelling and levels of acute phase reactants, demonstrating the responsiveness of the IC to immune modulation [60]. Moreover, evaluation of brain parameters in healthy volunteers in whom an intravenous injection of LPS induced systemic inflammation revealed that a stronger sickness response to LPS was significantly associated with a larger anterior insula gray matter volume [61]. However, the correlation was independent of cytokine levels, further highlighting a gap in our understanding regarding the nature of the specific information recorded by the IC in response to peripheral immunity.

Studies in mice provided more direct evidence for the involvement of the IC in immune activity. The first line of evidence comes from immune conditioning studies. Conditioning of immune functions was first demonstrated by Metalnikov & Chorine. They injected guinea pigs with the plant extract Tapioka (serving as the unconditioned stimulus, US), which increased peripheral leucocyte numbers. Together with the injection, the skin of the animals was either heated or slightly slit (the conditioned stimulus, CS). After several CS–US pairings, skin stimulation alone was sufficient to increase the leucocyte numbers, indicating a conditioned immune response. Despite its promise, this line of research was abandoned for over 50 years until Ader & Cohen demonstrated behaviorally conditioned immunosuppression employing a conditioned taste aversion paradigm in rats [62]. By employing the immunosuppressive drug cyclosporin A as a US in a taste aversion paradigm in rats, conditioned immunosuppression could be repeatedly demonstrated, reflected by a reduction in spleen and thymus weight [63], a reduced proliferation rate of lymphocytes in the spleen [64], and decreased interleukin-2 (IL-2) and γ-interferon (γ-IFN) levels [65]. Other studies demonstrated a conditioned enhancement of the immune responses [66], including a conditioned increase in natural killer cell (NK) activity in mice by pairing the odor of camphor with an injection of poly I:C, which stimulates NK activity. The role of the IC in these conditioning paradigms was directly tested, mainly by lesion studies [20, 22, 67, 68]. For example, the effect of lesions in the IC on the acquisition (lesions made before conditioning) or evocation (lesions made after conditioning) of a conditioned immunosuppression task was tested using a single pairing of saccharin taste and the immunosuppressive drug cyclophosphamide. The results showed that IC lesions disrupted both the acquisition and evocation of conditioning [20, 22, 67, 68].

We recently used the TRAP mice described above to express an excitatory form of DREADD in IC neurons active during inflammation (DSS-induced colitis, or zymosan-induced peritonitis) [18]. This paradigm enabled us to reactivate, following recovery, only the specific neuronal ensembles active during the original inflammation. The reactivation resulted in the induction of an inflammatory response at the same site as the original inflammation (colon or peritoneum). Moreover, inhibition of IC activity during DSS-induced colitis attenuated the inflammatory response, demonstrating the involvement of IC in immune modulation.

### Anatomical evidence

Although the capacity to monitor, record, and regulate immune activity is not limited to a specific brain area, the IC stands out as an especially relevant site for immune modulation. This is due in part to its anatomical connections to the peripheral sensory and autonomic nervous systems.

Interoceptive signals can be conveyed to the brain by sensory afferent pathways and humoral messengers that can be directly sensed by central neurons and glia. For example, osmoreceptors and glucoreceptors expressed by cells in the circumventricular organs (CVO), sites with a fenestrated blood‒brain barrier, can directly monitor changes in the blood. Similarly, cytokines and other inflammatory humeral signals can be sensed in CVOs. The direct sensing of humeral information is not a unique property of the IC; however, the anatomical inputs of sensory neurons indicate that the IC is a potential site of immune and interoceptive integration. The IC is heavily interconnected with various brain regions, including the somatosensory cortex [31], which processes sensory and interoceptive information from the body.

Peripheral inflammation induces afferent neural signals that can converge through the sensory nerves comprising the vagus nerve and the DRGs. These inputs reach several brain areas, most notably the nucleus tractus solitarius (NTS), the classical visceral receiving area in the brainstem. The NTS has numerous ascending projections to the hypothalamus, amygdala, striatum, cingulate cortex, and IC [69]. Assuming that these are functional sensory inputs that also reach the IC, an important open question is which sensory fibers detect the immune information and what is the nature of the relevant receptors. Moreover, we do not know what kind of immune-related information is detected by these sensory pathways.

Another attractive property of the IC as a potential site of immunoception is somatotopic organization, namely, the correspondence of an area of the body to a specific point on the brain. This concept is well known in the primary somatosensory cortex, typically represented as a sensory homunculus that orients the specific body parts and their respective locations upon the homunculus. Different levels of somatotopic organization are found in different brain systems, and in the IC, at least some sensory interoceptive afferents are somatotopically organized from posterior to mid to anterior [70]. This is relevant, as it represents the potential capacity of the IC to encode specific anatomical locations of inflammation.

The IC is also connected to outputs from the brain, mainly the autonomic nervous system [70, 71]. The NTS has lateral projections through intermediaries to vagal motor neurons in the dorsal motor nucleus of the vagus (DMV) and the nucleus ambiguus, as well as the rostral ventrolateral medulla (RVLM), which represent the efferent limbs of the pathway. Stimulation of the mouse RVLM or the mouse vagal efferents results in the suppression of innate immune responses and downregulation of proinflammatory cytokines in the spleen via a cholinergic mechanism [72].

Using retrograde labeling, we recently showed an anatomical connection between IC neurons that were active during peripheral inflammation (zymosan-induced peritonitis) and the RVLM and DMV. We injected the retrograde virus into the site of inflammation, the peritoneum, and used TRAP mice to capture the active neurons during inflammation [18]. We then expressed AAV1 virus, known to have anterograde propagation [73] in these active (TRAPed) neurons. This approach allowed us to visualize the anatomical site where the retrograde projections from the peritoneum meet the anterograde projections from the IC. Interestingly, the meeting point was in the two main autonomic output sites in the brain stem, the RVLM and the DMV, which, as indicated above, control the parasympathetic and sympathetic outputs. Moreover, retrograde anatomical mapping from different immune sites, including the spleen and bone marrow, demonstrated the anatomical connection to the IC [74]. These studies manifest the unique position of the IC as a site potentially able to control the peripheral immune system, specifically via the sympathetic and parasympathetic systems. These are likely to be functional connections, as in patients with acute ischemic stroke, autonomic dysfunction has been associated with worse outcomes, including immune depression [75]. In these studies, the involvement of the IC is suspected to play a significant role in causing sympathovagal imbalance.

Taken together, these lines of evidence suggest that the IC plays a role in forming the brain’s representation of the immune state and in regulating immunity. However, it is important to note that the neuroimmune dialog extends beyond the IC, and it is likely that many brain networks participate in immunoception. The IC stands out due to its unique combination of features. It possesses anatomical connections that enable communication with the peripheral immune system, specializes in processing interoceptive signals relevant to immune regulation, and integrates multiple sensory modalities. These characteristics position the IC as a central hub for interoception in the brain and potentially also for immunoception.

## Insular cortex immunity and pain

As mentioned, the IC is also part of the pain matrix [28]. Pain and immunity are closely connected, as pain can be a consequence of inflammation and immune activation, and the immune system can also modulate pain sensitivity and perception [76, 77]. Proinflammatory cytokines sensitize pain-related receptors and increase their responsiveness to noxious stimuli (e.g., heat, pressure, chemicals) [78]. This phenomenon is known as peripheral sensitization, and it can also affect the central nervous system, leading to central sensitization, which is characterized by increased excitability of the neurons in the spinal cord and in brain regions involved in pain processing (e.g., thalamus, prefrontal cortex, IC) [76]. These central effects control pain signaling, as well as the activity of the descending pain pathways that originate in the brain and modulate pain perception at the spinal level. Other mechanisms through which the immune system can influence pain sensitivity and perception are through the modulation of microglial cells or changes in the expression of ion channels and receptors in sensory neurons [79].

On the other hand, pain can also affect immunity. Chronic pain was shown to lead to changes in immune activity, mostly immune suppression [80], for example, via the hypothalamic‒pituitary‒adrenal (HPA) axis and sympathetic nervous system, which are modulated by chronic pain.

Multiple studies have shown that the insula is critical in perceiving pain intensity, quality, and location [28, 32, 40, 79, 81]. For example, neuroimaging studies have demonstrated that the IC shows increased activation during the experience of pain and that this activation correlates with the subjective ratings of pain intensity and unpleasantness [81]. Moreover, the IC has been shown to be involved in the emotional and motivational aspects of pain, such as fear, anxiety, and empathy, as well as in the cognitive processes related to pain modulation, such as attentional bias and pain coping strategies [82]. These studies raise the possibility that part of the immune information encoded by the brain is pain-related. Indeed, we TRAPed neurons in the IC during zymosan-induced peritonitis in the presence of analgesia [1]. Interestingly, reactivation of the same neuronal ensembles resulted in an immune outcome that was different from the one observed when we reactivated the trace captured without analgesia, suggesting the potential relevance of pain as part of the immune-related information encoded by the IC. Nevertheless, it is important to note that our understanding of the fundamental connections between pain and immunity remains very limited. Moreover, although these lines of evidence support the involvement of the IC in immunoception, they also highlight that we are still in the early stages of this research. One critical question is how immune information is represented by the brain and what kind of information is stored by the IC.

## Immune engram

The neuronal representation of the immune state by the brain, specifically by the IC, suggests that there is a specific neuronal trace that captures the immune information, an immunengram [1]. The classical neuroscience concept of an engram refers to the neural substrate underlying the storage and retrieval of memories, namely, the physical representation of memories in the brain [83]. Although the exact nature of the engram is still a topic of active research and debate, it is generally accepted that memories are stored as patterns of activity across networks of neurons rather than in isolated individual cells. The engram is not limited to a single modality and can be affected by various inputs. For example, a recent study demonstrated in mice that long-term associative fear memory stored in neuronal engrams in the prefrontal cortex determines whether a painful episode shapes pain experience later in life [84]. Under conditions of neuropathic pain, prefrontal fear engrams expand to encompass neurons representing nociception and tactile sensation, leading to pronounced changes in prefrontal connectivity to fear-relevant brain areas. This highlights the complexity of engrams at the brain level. However, an immunengram is expected to have additional, unique properties.

The concept of an immunengram suggests that the brain can form a specific neuronal trace in response to immunological events, which can be retrieved upon reactivation of the same neuronal ensembles. However, in contrast to the neuronal engram, which is specific to the brain, and for which neuronal activity is sufficient to manifest the required behavior, we suggest that in the immunengram, the trace is not limited to the neuronal component. It involves changes in tissue cells in the periphery and, potentially, in specific immune clones [1]. Such a distributed trace is necessary because the immune system operates as a complex and distributed network of cells and molecules. The brain can communicate with the immune system through a limited set of tools, mainly the autonomic nervous system. By forming a distributed trace, which involves changes in both neuronal circuits and peripheral tissue components, the brain can better communicate with and regulate the immune response in peripheral tissues. Tissue components, such as immune cells and neuropeptide receptors, can act as interpreters of the limited peripheral neuronal input and eventually recapitulate part of the complexity of the tissue’s previous inflammatory event. In other words, the distributed trace allows for more nuanced and adaptable communication between the brain and the immune system, which is crucial for effective immune regulation and response.

## Summary

Here, we present some emerging evidence indicating that immune-related information is stored in the brain and that the brain uses such information to orchestrate physiological processes and regulate immune activity. However, many open questions remain in the field. Thus, for example, it is not known what kind of immune information is acquired by the brain, how it is conveyed to the CNS, which brain areas and neuronal netrworks record this information, how this information is integrated with previously available inputs, whether the brain can update the stored information, and how the brain executes its control over the immune response. Nevertheless, the potential impact of such brain representation and regulation of immunity on our understanding of physiology is enormous. These concepts challenge the common perception of immunological memory as stored solely by the immune system to include “immune memory” by the nervous system. This further suggests that autoimmune disease can be triggered by neuronal stimuli, providing new mechanistic insights into psychosomatic disorders. Moreover, it can pave the way for a novel potential therapeutic modality regulating immunity by manipulating the brain.
